# Rigid Shape Registration Based on Extended Hamiltonian Learning

**DOI:** 10.3390/e22050539

**Published:** 2020-05-12

**Authors:** Jin Yi, Shiqiang Zhang, Yueqi Cao, Erchuan Zhang, Huafei Sun

**Affiliations:** 1 Department of Basic Courses, Beijing Union University, Beijing 100081, China; yijin@buu.edu.cn; 2 School of Mathematics and Statistics, Beijing Institute of Technology, Beijing 100081, China; 3120181406@bit.edu.cn (S.Z.); 3120181396@bit.edu.cn (Y.C.); 3 School of Mathematics and Statistics, University of Western Australia, Crawley WA6009, Australia; erchuan.zhang@research.uwa.edu.au

**Keywords:** rigid registration, iterative closest point, special Euclidean group, extended Hamiltonian learning, 53B20, 68U10

## Abstract

Shape registration, finding the correct alignment of two sets of data, plays a significant role in computer vision such as objection recognition and image analysis. The iterative closest point (ICP) algorithm is one of well known and widely used algorithms in this area. The main purpose of this paper is to incorporate ICP with the fast convergent extended Hamiltonian learning (EHL), so called *EHL-ICP algorithm*, to perform planar and spatial rigid shape registration. By treating the registration error as the potential for the extended Hamiltonian system, the rigid shape registration is modelled as an optimization problem on the special Euclidean group SE(n) (n=2,3). Our method is robust to initial values and parameters. Compared with some state-of-art methods, our approach shows better efficiency and accuracy by simulation experiments.

## 1. Introduction

Point set registration is to find an optimal transformation that aligns one set to the other. This problem occurs in many applications, such as motion tracking [1,2], target localization [3], super-resolution [4], mosaicing [5] and medical image analysis [6,7,8,9,10]. Depending on different goals, the methods can be categorized as coarse registration and fine registration [11,12]. The former is to find an initial estimation between two point sets while the latter is to obtain a more accurate solution. In general, the popular method used in coarse registration is point-to-point and iterative, such as point signature and spin image. Some other methods may include principle component analysis (PCA), algebraic surface model and principle curvature. While methods like iterative closed point (ICP), Chen’s method, signed distance fields and genetic algorithms, are more commonly used in fine registration.

Literately, the ICP algorithm is efficient and accurate among all point set registration techniques. However, it requires that the initial configuration of the two point sets is sufficiently close and it is sensitive to noise and outliers. Moreover, the ICP algorithm is unable to handle affine case. To improve the robustness and cope with more situations, many researchers extended the ICP method. In particular, Ying et al. applied the theory of differential geometry and Lie group to ICP algorithm and formed a unified framework to design fast and robust shape registration algorithms [13,14,15,16,17,18,19,20,21].

From the geometric point of view, given the correspondence between two sets of points, the affine registration is to find an element in the general linear group that transfers one set to the other optimally. In this paper, we deal with the rigid registration, which uses the geometry of the special Euclidean group. From this perspective, differential geometry is a powerful tool not only in registration problems but also in computer vision extensively. There is a vast community dealing with shape registration/analysis/comparison from geometrical viewpoints. For example, E. Celledoni etc. applied the theory of Lie groups and homogeneous manifolds to the problem of shape analysis [22,23]. A.L. Brigant introduced an algorithm that finds an optimal matching between two curves by computing the geodesic of the infinite-dimensional manifold of curves [24]. X. Pennec etc. concentrated on feature-based approaches for rigid registrations using differential geometry of surfaces [25]. Other methods, using harmonic analysis and statistical optimizations, can be found in [26,27,28,29]. Experiments showed that these geometry-based approaches performed better than other state-of-art methods.

There is also an immense literature in applying Hamiltonian dynamical systems to learning theory. F. Barbaresco studied the symplectic extension of Souriau Lie groups thermodynamics and used this model for data analysis and machine learning on Lie groups [30,31]. S. Fiori proposed extended Hamiltonian learning (EHL) on Riemannian manifolds motivated by Hamilton variational principle [32,33]. Compared with the classical gradient-based learning, EHL has the advantages of averaging out the oscillations and mitigating the plateau effect. In this paper, we investigate the 2D/3D rigid registration problem from the view of Hamiltonian learning. The innovation of our proposed method is treating the registration error as the potential of the extended Hamiltonian system on the underlying space of transformation, the special Euclidean group. Under the system of extended Hamiltonian, an EHL-based algorithm is designed to achieve the optimal transformation corresponding to the minimum of the registration error. Some experiments are carried out to validate the efficiency and robustness of our algorithm.

The structure of the paper is organized as follows. In Section 2, some basic background about geometric structures of the special Euclidean group SE(2) and SE(3) is reviewed, including the Riemannian metric, geodesic, and Riemannian gradient. In Section 3, extended Hamiltonian learning on a general Riemannian manifold is discussed. And in the following section, we formulate the method for 2D/3D rigid registration problem by extended Hamiltonian learning on SE(2) and SE(3). In Section 5, some numerical experiments prove the efficiency of this proposed method, comparing with Du’s SVD method (SVD) [16], Ying’s Iwasawa decomposition method (ID) [21], Ying’s Lie group optimization method (LGO) [18]. At last, conclusions are presented and possible improvements of our method for future work are discussed.

## 2. Geometry of Special Euclidean Groups

In this section, we review some basics about the special Euclidean group SE(n), which is the underlying geometric space for rigid registration. The special Euclidean group consists of rotations and translations in the Euclidean space. It is the semi-direct product of the special orthogonal group SO(n) and $R^{n}$,

(1) $$SE\left(n\right)=SO\left(n\right)⋉R^{n}.$$

Being a Lie group, SE(n) is equipped with the smooth structure and group structure simultaneously.

Any element *g* in SE(n) can be represented as a pair (r,t), where r∈SO(n) and $t\in R^{n}$ stands for the rotation and translation respectively. In the matrix form, *g* can be written as

(2) $$\begin{array}{c} g=\left(\begin{array}{cc} r & t\\ 0 & 1 \end{array}\right), \end{array}$$

which is an one-to-one correspondence with the pair (r,t). The action of the special group SE(n) on the Euclidean space is defined as

(3) $$\begin{array}{c} \left(\begin{array}{cc} r & t\\ 0 & 1 \end{array}\right)\cdot \left(\begin{array}{c} y\\ 1 \end{array}\right)=\left(\begin{array}{c} ry+t\\ 1 \end{array}\right). \end{array}$$

The Lie algebra of SE(n), denoted by se(n), contains infinitesimal rotations and translations. A general element *h* in se(n) is a pair (J,v), where *J* is a n×n skew-symmetric matrix and *v* is a vector in $R^{n}$. In matrix form, *h* can be rewritten as

(4) $$\begin{array}{c} h=\left(\begin{array}{cc} J & v\\ 0 & 0 \end{array}\right)\in \mathrm{se}\left(n\right). \end{array}$$

The exponential mapping and the logarithm mapping provide methods to transfer information between the nonlinear manifold SE(n) and the linear space se(n). Concretely, the exponential map of a general Lie algebra element is defined by

(5) $$\begin{array}{cc} \exp:\mathrm{se}(n) & \to SE(n)\\ h & \mapsto \sum_{n=0}^{\infty }\frac{1}{n!}{\left(\begin{array}{cc} J & v\\ 0 & 0 \end{array}\right)}^{n}. \end{array}$$

In low dimensions the exponential map can be written in a compact form, as presented by J.M. Selig [34]. Denote $\omega =\sqrt{\omega_{x}^{2}+\omega_{y}^{2}+\omega_{z}^{2}}$, where

(6) $$\begin{array}{c} J=\left(\begin{array}{ccc} 0 & -\omega_{z} & \omega_{y}\\ \omega_{z} & 0 & -\omega_{x}\\ -\omega_{y} & \omega_{x} & 0 \end{array}\right). \end{array}$$

For any h∈se(3), direct computation shows that it satisfies the quartic equation $h^{4}+\omega^{2}h^{2}=0$. Thus the higher order terms in the taylor expansion (5) can be simplified. Explicitly, we have

(7) $$\exp\left(h\right)=I_{4}+h+\frac{\left(1-\cos\omega \right)}{\omega^{2}}h^{2}+\frac{\left(\omega -\sin\omega \right)}{\omega^{3}}h^{3}.$$

Especially, for pure rotations, where v=0 in the expression (4) and *h* satisfies the cubic equation $h^{3}+\omega^{2}h=0$, the exponential map can be written as

(8) $$\exp\left(h\right)=I_{4}+\frac{\sin\omega }{\omega }h+\frac{\left(1-\cos\omega \right)}{\omega^{2}}h^{2},$$

while for pure translations, where J=0 in the expression (4) and $h^{2}=0$, the exponential map satisfies

(9) $$\exp\left(h\right)=I_{4}+h.$$

The exponential map for pure rotation degenerates on se(2) to be as the following,

(10) $$\exp\left(h\right)=\left(\begin{array}{cc} \left(\begin{array}{cc} \cos\omega & -\sin\omega \\ \sin\omega & \cos\omega \end{array}\right) & 0\\ 0 & 1 \end{array}\right),h=\left(\begin{array}{ccc} 0 & -\omega & 0\\ \omega & 0 & 0\\ 0 & 0 & 0 \end{array}\right)\in \mathrm{se}\left(2\right).$$

An inner product on the Lie algebra g can be extended to a Riemannian metric on the Lie group *G* by left translation. Specifically, the inner product at se(n) is defined by

(11) $$\begin{array}{c} {\langle \left(J_{1},v_{1}\right),\left(J_{2},v_{2}\right)\rangle }_{I}=m\mathrm{Trace}\left(J_{1}^{T}J_{2}\right)+v_{1}^{T}v_{2}, \end{array}$$

where m>0 is a fixed parameter, $\left(J_{1},v_{1}\right),\left(J_{2},v_{2}\right)\in \mathrm{se}\left(n\right)$. The left-invariant metric, for two tangent vectors $\alpha_{1}$ and $\alpha_{2}$ at an arbitrary group element g∈SE(n) is defined as

(12) $$\begin{array}{c} {\langle \alpha_{1},\alpha_{2}\rangle }_{g}={\langle g^{-1}\alpha_{1},g^{-1}\alpha_{2}\rangle }_{I}. \end{array}$$

The right-invariant metric is defined similarly. In physics, the left-invariance and right-invariance correspond to the independence of the choices of the inertial frame and the body-fixed frame respectively [35]. A bi-invariant metric means it is both left-invariant and right-invariant. However, there is no bi-invariant metric on SE(n) [36]. Thus, we adopt the left-invariant metric on SE(n) throughout this paper.

Let *f* be a function defined on Riemannian manifold *M*, $\partial_{x}f$ and $\nabla_{x}f$ be Euclidean gradient and Riemannian gradient, respectively. The relation between $\partial_{x}f$ and $\nabla_{x}f$ is governed by [32]

(13) $$\begin{array}{c} {\langle \partial_{x}f,u\rangle }_{E}={\langle \nabla_{x}f,u\rangle }_{R},\forall u\in T_{x}M, \end{array}$$

where ${\langle \cdot ,\cdot \rangle }_{E}$ and ${\langle \cdot ,\cdot \rangle }_{R}$ denote Euclidean inner product and Riemannian metric respectively. For the Riemannian metric defined in (12) on SE(n), we have

(14) $$\begin{array}{c} \left\{\begin{array}{c} \nabla_{r}f=\frac{1}{2m}\left(\partial_{r}f-r{\left(\partial_{r}f\right)}^{T}r\right)\\ \nabla_{t}f=\partial_{t}f \end{array}\right.. \end{array}$$

## 3. Extended Hamiltonian Learning on SE(n)

We first introduce the extended Hamilton learning on general Riemannian manifolds [32,33]. Then we specify on special Euclidean group SE(n).

On a Riemannian manifold *M*, equipped with a metric ${\langle \cdot ,\cdot \rangle }_{x}$ at $T_{x}M$, the extended Hamiltonian principle is

(15) $$\begin{array}{c} \delta \int_{t_{1}}^{t_{2}}\left(K_{x}\left(\dot{x},\dot{x}\right)-V\right)dt+\int_{t_{1}}^{t_{2}}{\langle f_{x},\delta x\rangle }_{x}dt=0. \end{array}$$

In Equation (15), x(t)∈M stands for the trajectory of a particle moving along *M* and $\dot{x}\left(t\right)$ is the corresponding instantaneous velocity. The function $K:T_{x}M\times T_{x}M\to R$ denotes the kinetic energy and *V* represents associated potential energy. The symbol δ represents the variation of the action in the dynamical system, namely, the particle slides from a point to an infinitely close point. An element $f_{x}\in T_{x}M$ indicates the dissipation force at the point *x*. This system degenerates to conservative if the dissipation force disappears everywhere. Following [32,33], the kinetic energy is adopted as the symmetric bilinear form $K_{x}\left(\dot{x},\dot{x}\right)=\frac{1}{2}{\langle \dot{x},\dot{x}\rangle }_{x}$ under the assumption of unit mass. From viscosity theory the dissipation term is assumed to be $f_{x}=-\mu \dot{x}$ with μ≥0 in the paper. Then, the equations for such dynamical system read

(16) $$\begin{array}{c} \left\{\begin{array}{c} \dot{x}=v,\\ \dot{v}=-\Gamma_{x}\left(v,v\right)-\nabla_{x}V-\mu v, \end{array}\right. \end{array}$$

where $\Gamma_{x}$ is the Christoffel symmetric form, and $\nabla_{x}V$ denotes the Riemannian gradient of *V*.

In order to implement simulations, we turn the continuous version (16) into a discrete one, which yields

(17) $$\begin{array}{c} \left\{\begin{array}{c} x_{k+1}=R_{x_{k}}\left(\eta v_{k}\right),\\ v_{k+1}=\left(1-\eta \mu \right)v_{k}-\eta \left(\Gamma_{x_{k}}\left(v_{k},v_{k}\right)+\nabla_{x_{k}}V\right), \end{array}\right. \end{array}$$

where $R_{x_{k}}:T_{x_{k}}M\to M$ is the exponential map and η>0 is the selected step size.

When it comes to the special Euclidean group SE(n), we need to compute the Christoffel symmetric form. However, in the discrete case, it suffices to know the form in a subspace so(n) of se(n). Specifically, the Christoffel symmetric form for g=(r,t)∈SE(n), h=(J,0)∈se(n) reads

(18) $$\begin{array}{c} \Gamma_{g}\left(gh,gh\right)=\left(-rJ^{2},0\right). \end{array}$$

Then Equation (17) can be rewritten as

(19) $$\begin{array}{c} \left\{\begin{array}{c} r_{k+1}=R_{r_{k}}\left(\eta r_{k}J_{k}\right)=r_{k}R_{I}\left(\eta J_{k}\right)\\ r_{k+1}J_{k+1}=\left(1-\eta \mu \right)r_{k}J_{k}-\eta \left(\nabla_{r_{k}}V-r_{k}J_{k}^{2}\right) \end{array}\right., \end{array}$$

**Figure 1:**
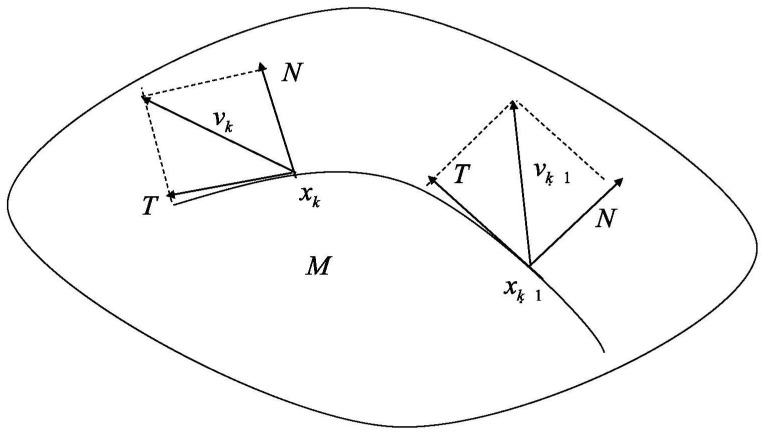
Iteration with decomposition of $v_{k+1}$.

Note that from the iteration (19) one cannot ensure that $J_{k+1}$ is skew-symmetric. Consequently $r_{k+1}$ will not be an element in SE(n). Thus, $J_{k+1}$ should be modified to keep the validity of the iteration. To do this we make an orthogonal projection from the Euclidean space $R^{n^{2}}$ to the subspace consisting of skew-symmetric matrices. i.e., For any matrix $J_{k+1}$, it can be decomposed as the sum of a skew-symmetric part and a symmetric part $J_{k+1}=\frac{1}{2}\left(J_{k+1}-J_{k+1}^{T}\right)+\frac{1}{2}\left(J_{k+1}-J_{k+1}^{T}\right)$. From (19), the symmetric part $\frac{1}{2}\left(J_{k+1}-J_{k+1}^{T}\right)$ would be a negligible error when the step is small enough. Thus we apply the skew-symmetric part in the tangent space $T_{r_{k+1}}SE(n)$. See Figure 1. We remark that this step is nothing but the to compute the covariate derivative on Euclidean submanifolds by definition [37]. Hence, the iteration becomes

(20) $$\begin{array}{c} \left\{\begin{array}{c} r_{k+1}=r_{k}R_{I}\left(\eta J_{k}\right)\\ J_{*}=r_{k+1}^{-1}\left(\left(1-\eta \mu \right)r_{k}J_{k}-\eta \left(\nabla_{r_{k}}V-r_{k}J_{k}^{2}\right)\right)\\ J_{k+1}=\frac{1}{2}\left(J_{*}-J_{*}^{T}\right) \end{array}\right.. \end{array}$$

For details about the convergence of extended Hamiltonian learning, refer to [32]. The necessary condition for convergence is that μ satisfies $\sqrt{2\lambda_{\max}}<\mu <\eta^{-1}$, where $\lambda_{\max}$ is the maximum eigenvalue of the Hessian matrix of *V*.

## 4. 2D/3D Rigid Shape Registration Based on Extended Hamiltonian Learning

Given two n-D (n = 2,3) data sets $X={\left\{x_{i}\right\}}_{i=1}^{N_{x}}$ and $Y={\left\{y_{j}\right\}}_{j=1}^{Ny}$ and a correspondence $\pi :\left\{1,\cdots ,N_{x}\right\}\to \left\{1,\cdots ,N_{y}\right\}$, denoting $z_{i}=y_{\pi (i)}$, find an element g=(r,t)∈SE(n) such that the cost function

(21) $$\begin{array}{c} V\left(g\right)=\frac{1}{N_{x}}\sum_{l=1}^{N_{x}}||rx_{l}+t-z_{l}{\left|\right|}^{2} \end{array}$$

achieves it minimum.

The fundamental steps based on ICP for rigid registration are: First, for the current fixed transformation $g^{k}=\left(r^{k},t^{k}\right)\in SE\left(n\right)$, find $Z^{k}\subseteq Y$ with $N_{x}$ elements such that the subset minimizes

(22) $$\begin{array}{c} V\left(Z\right)=\frac{1}{N_{x}}\sum_{l=1}^{N_{x}}\left|\right|r^{k}x_{l}+t^{k}-z_{l}{\left|\right|}^{2},Z\subset Y. \end{array}$$

Second, once the correspondent data set $Z^{k}$ is obtained, update the transformation $g^{k}$ as $g^{k+1}$ for minimizing

(23) $$\begin{array}{c} V\left(g\right)=\frac{1}{N_{x}}\sum_{l=1}^{N_{x}}{∥rx_{l}+t-z_{l}∥}^{2},g=\left(r,t\right)\in SE\left(n\right). \end{array}$$

In fact, the translation *t* can eliminated by coordinating the centers of the two data sets. Let $x_{c}$ and $y_{c}$ be centers of *X* and *Y*, respectively. For the obtained set $Z^{k}$, the least squares solution $\left(r^{k+1},t^{k+1}\right)$ of (23) indicates

(24) $$\begin{array}{c} t^{k+1}=y_{c}-r^{k+1}x_{c}. \end{array}$$

Thus, with the centralized data ${\tilde{x}}_{i}=x_{i}-x_{c}$, optimization problems (22) and (23) can be simplified as

(25) $$\begin{array}{cc} Z^{k} & =\underset{Z\subset Y}{\arg\min}V\left(Z\right)=\underset{Z\subset Y}{\arg\min}\frac{1}{N_{x}}\sum_{l=1}^{N_{x}}∥r^{k}{\tilde{x}}_{l}+t^{k}-z_{l}{)∥}^{2}\\ r^{k+1} & =\underset{r\in SO(n)}{\arg\min}V\left(r\right)=\underset{r\in SO(n)}{\arg\min}\frac{1}{N_{x}}\sum_{l=1}^{N_{x}}{∥r{\tilde{x}}_{l}+t^{k}-z_{l}^{k}∥}^{2}. \end{array}$$

Here, we regard the registration error as potential of extended Hamiltonian system on the special Euclidean group SE(n). The Euclidean gradient of V(r) is given as

(26) $$\begin{array}{c} \partial_{r}V\left(r\right)=\frac{2}{N_{x}}\sum_{l=1}^{N_{x}}{\tilde{x}}_{l}{\left(r{\tilde{x}}_{l}+t^{k}-z_{l}^{k}\right)}^{T}, \end{array}$$

from which we can compute the Riemannian gradient of V(r) by (14).

Therefore, for two given data sets $X={\left\{x_{i}\right\}}_{i=1}^{N_{x}}$ and $Y={\left\{y_{j}\right\}}_{j=1}^{N_{y}}$, we summarize the method based on extended Hamiltonian learning as Algorithm 1.

**Algorithm 1** EHL-ICP Algorithm**Input:** Initial Data ${\left\{x_{i}\right\}}_{i=1}^{N_{x}}$; Target Data ${\left\{y_{i}\right\}}_{i=1}^{N_{y}}$ **Output:** Rotation $r^{k}$; Translation $t^{k}$; Registration Error $V^{k}$;
 1:Initialize parameters $r^{0}$, $J^{0}$, η,μ,ϵ>0; 2:Set $x_{c}=\frac{1}{N_{x}}\Sigma x_{i}$, $y_{c}=\frac{1}{N_{y}}\Sigma y_{i}$; 3:Centralize $x_{i}$ as ${\tilde{x}}_{i}=x_{i}-x_{c}$, $i=1,\cdots ,N_{x}$, and set $t^{0}=y_{c}-r^{0}x_{c}$; 4:**for**k=0,⋯**do** 5:    Search for $z^{k}$ by minimizing $\sum_{l=1}^{N_{x}}\left|\right|r^{k}{\tilde{x}}_{l}+t^{k}-z_{l}{\left|\right|}^{2}$; 6:    Calculate the registration error $V^{k}=\frac{1}{N_{x}}\sum_{l=1}^{N_{x}}\left|\right|r^{k}{\tilde{x}}_{l}+t^{k}-z_{l}^{k}{\left|\right|}^{2}$; 7:    Search for $r^{k+1}$ by minimizing $V\left(r\right)=\frac{1}{N_{x}}\sum_{l=1}^{N_{x}}||r{\tilde{x}}_{l}+t^{k}-z_{l}^{k}{\left|\right|}^{2}$ by Equation (19); 8:    $t^{k+1}=y_{c}-r^{k+1}x_{c}$; 9:    **if** $(1-V^{k}/V^{k-1})\leq \epsilon$ **then**10:        **return** $\left(r^{k},t^{k},V^{k}\right)$11:    **else**12:        k=k+1;13:    **end if**14:**end for**


## 5. Numerical Results

All data samples used in this section is from the MPEG-7 shape B database and all programs are written in Matlab 2018a and run by PC with AMD Athlon II P340 Dual-Core processor, 2.20 GHz CPU, and 2 GB RAM.

### 5.1. 2D Rigid Shape Registration

**Figure 2:**
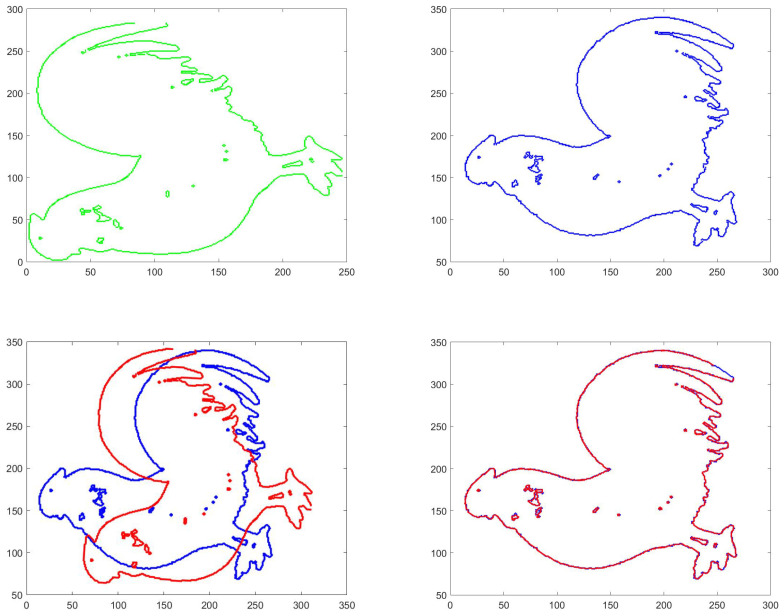
The (**top left**) figure is the model data, the (**top right**) figure is the test data, the (**bottom left**) is the figure after five iterations and the (**bottom right**) is the figure after final registration.

In 2D case, our method appears to be robust and insensitive to initial values. Moreover, a near-optimal registration can be obtained within a few steps. To give a visualization of our method, we test chicken-2 and chicken-3 in MPEG-7 shape B database as model data set and test data set. The initial rotation is set to be the identity and the initial translation is set to be the difference of two means of data. The numerical results are shown in Figure 2.

We select nine groups of rigid data in MPEG-7 shape B database to run the experiments and compare with Du’s SVD method, Ying’s Iwasawa decomposition method and Ying’s Lie group optimization method. To give a quantitive comparison we define the root mean square (RMS) error to be

(27) $$\mathrm{RMS}={\left(\frac{1}{n}\sum_{i=1}^{n}{\left(X_{i}-Y_{i}\right)}^{2}\right)}^{\frac{1}{2}}$$

for model data ${\left\{X_{i}\right\}}_{i=1}^{n}$ and test data ${\left\{Y_{i}\right\}}_{i=1}^{n}$. Though the geodesic distance on SE(2) is more reasonable in theory, practically it is difficult to compute since we do not know the true rigid transformation.

**Figure 3:**
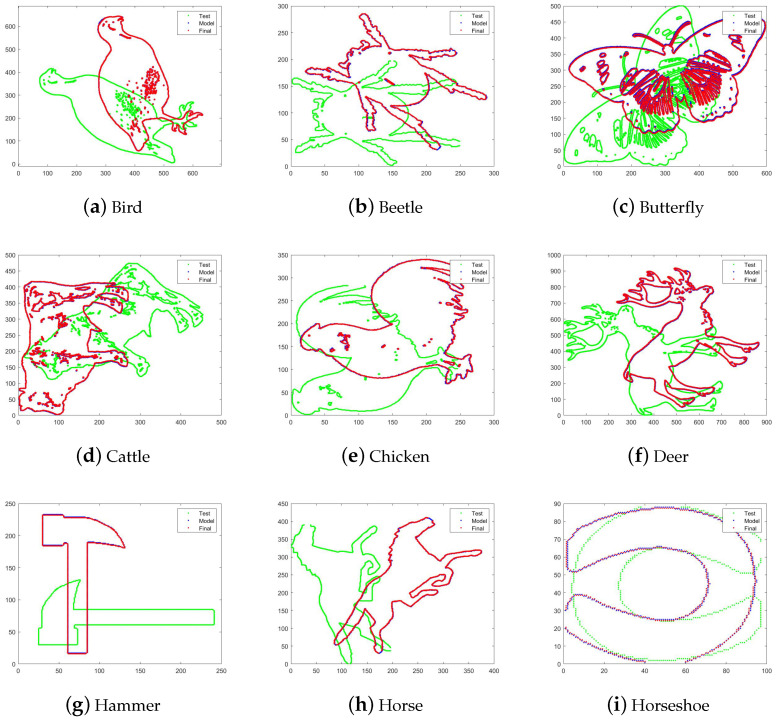
Experiments using different data sets. The test data are colored green; the model data are colored blue; the registration results are colored red.

The precision is set to be ϵ=0.00001. Comparison results are displayed in Figure 3. The resulted RMS errors are displayed in Table 1. Other methods require the careful choice of initial values, whereas for our method we simply choose identity as the initial rotation and difference of means as initial translation. We can find that EHL-ICP algorithm is more robust to the size and shape of point cloud data. Other algorithms may have good performance on small point sets with simple shapes but lose precision when point sets are complex.

### 5.2. 3D Shape Registration

**Figure 4:**
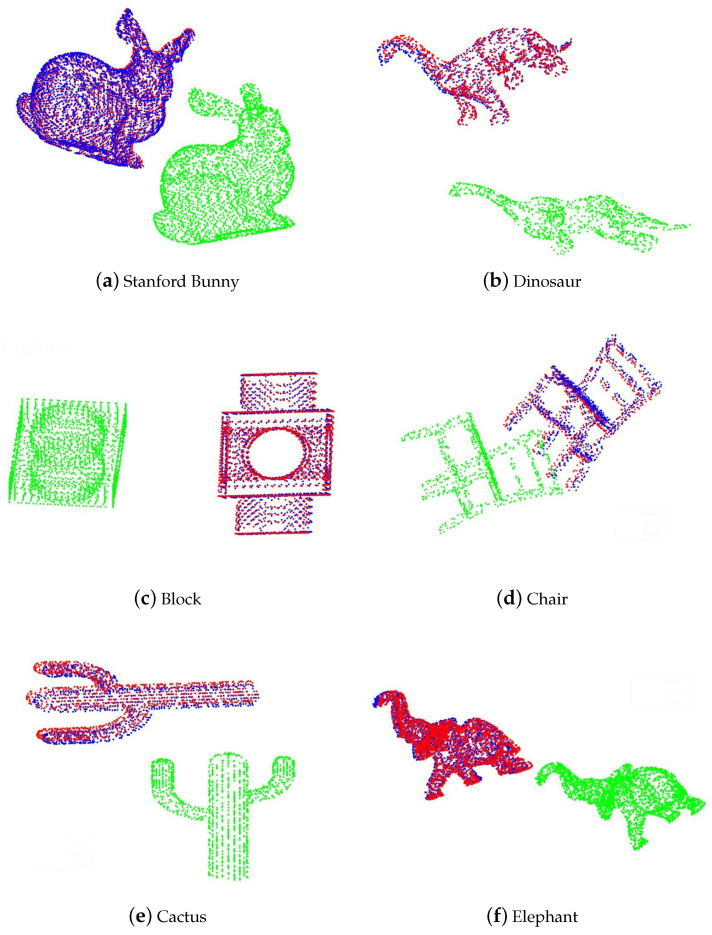
Blue: Model data (fixed); Green: Test data (moving); Red: Final data (registration). Figures of Stanford Bunny, dinosaur, block, chair, cactus, elephant.

Similar to the 2D case, we selected a group of 3D models including bunny, chair, cactus, dinosaur, elephant and block to verify the validity of our algorithm. The initial rotation is set to be identity and the initial translation is set to be the difference of means of model data and test data. The visualized results are represented in Figure 4. Note that we do not require a subtle choice of initial values and parameters. The results demonstrate the efficacy of our method.

## 6. Conclusions and Future Works

Shape registration plays a significant role in computer vision, where the task is to transfer one set of points to the other. Since the iterative closest point method is widely used in registration problems yet having some shortcomings, this paper proposes the EHL-ICP method, which incorporating the extended Hamiltonian learning with the ICP algorithm, to deal with the 2D and 3D rigid shape registration problem. By regarding the registration error as the potential of the extended Hamiltonian system, we formulate rigid registration as an optimization problem on the special Euclidean group SE(n) (n=2,3). Numerical results show that our method is more effective and accurate when compared with other methods. Moreover, our method is robust with respect to initial values in both dimensions, which provides a good choice for rough registration.

For future work, we may generalize the extended Hamiltonian learning method to different registration problems. There are two hot topics worth mentioning. The first is to use optimal transportation for data set registration [38,39]. The basic idea is that shape data can be viewed as a sum of Dirac measures in a given space and the difference of shapes is taken to be the Wasserstein distance. To find a (non)rigid transformation is to find a measure-preserving map. Another possible application is affine registration where shapes are distorted and there is no rigid transformation [40,41]. From this viewpoint, we should consider extended Hamiltonian learning on the general linear group GL(n), where more techniques should be developed.

## Figures and Tables

**Table 1:** Comparison of the RMS errors for 2D rigid shape registration. The optimal results are in bold.

Group	Model	Test	SVD	ID	LGO	EHL-ICP
(1)	bird-3	bird-4	0.5841	0.9996	0.5690	**0.4048**
(2)	deer-1	deer-4	**0.5263**	2.7598	0.5272	2.8826
(3)	horse-3	horse-4	0.5107	1.4278	0.5112	**0.3880**
(4)	beetle-7	beetle-8	0.8749	0.8746	0.5242	**0.4730**
(5)	cattle-1	cattle-20	22.8580	28.3719	22.5155	**1.1656**
(6)	hammer-4	hammer-5	0.4846	0.8037	0.4232	**0.3043**
(7)	chicken-2	chicken-3	0.5484	2.7 843	0.5471	**0.5202**
(8)	butterfly-1	butterfly-2	18.1726	34.3588	6.9691	**2.9062**
(9)	horseshoe-9	horseshoe-17	0.5425	0.5690	0.5873	**0.3577**
